# A Legal and Epidemiological Consideration of the Causal Relationship between Tobacco and Lung Cancer

**DOI:** 10.31557/APJCP.2021.22.9.2723

**Published:** 2021-09

**Authors:** Minsoo Jung

**Affiliations:** 1 *Takemi Fellow, Department of Global Health and Population, Harvard T.H. Chan School of Public Health, Boston, MA. *; 2 *Associate Professor, Department of Health Sciences, Dongduk Women’s University, Seoul, South Korea.*

**Keywords:** Causation, legal epidemiology, tobacco, lung cancer

## Abstract

This study reviewed issues including causality among tobacco companies’ illegal acts, smoking, and lung cancer occurrence. In tobacco lawsuits so far, the burden of proof regarding negligence and a causal relationship has fallen on plaintiffs, who are the injured party. However, since the legislation of the Product Liability Act, the possibility of mitigating plaintiffs’ burden of proof has opened up. Nevertheless, this alone cannot prevent the immense socioeconomic cost incurred due to smoking. It is legislatively necessary to enact a tobacco management law so that the no-fault liability of tobacco companies, which are the defendants, for compensation can be acknowledged. However, it is necessary to take supplementary measures through the social security system such as establishing the upper limits for liquidated damages in lawsuits and creating a relief fund for the victims of smoking. In addition, it is fundamentally necessary for courts to accept the methods for inferring causality that are based on the natural sciences and epidemiology in situations such as tobacco lawsuits, where a causal relationship cannot be proven easily. In particular, jurists, too, must consider the application of population-based evidence presented by epidemiologists to lawsuits in a forward-looking manner for redressing damages to individuals with diseases; thus, bridging the gap between normative adjudication and scientific judgment to draw a conclusion about a causal relationship.

## Introduction

In April 2015, the National Health Insurance Service (NHIS) in South Korea brought lawsuits against Philip Morris International (PMI) Korea, British American Tobacco (BAT) Korea, and Korea Tomorrow and Global (KT&G; Korean Tobacco Firm), which are tobacco companies, regarding claim for compensation for damages caused by smoking. The NHIS thus raised tobacco lawsuits in South Korea, which has a national health insurance (NHI) system, because smoking caused various diseases, which in turn led to a financial leakage in NHI due to the immense costs of treatment. Admittedly, the country had previously witnessed lawsuits raised by individuals with lung cancer or lung diseases against tobacco companies. However, because it was the first time for a state organ to bring lawsuits as a representative of public interest, the cases received public attention. The main contents of these lawsuits included a demand to the above mentioned tobacco companies of approximately 50 billion won which the NHIS had unnecessarily disbursed to 3,500 patients with a smoking history of more than 20 pack years and a smoking duration of more than 30 years among the cancer patients with specific carcinomas that were reported as likely to be caused by smoking (e.g., small cell lung cancer, squamous cell lung cancer, and squamous cell laryngeal cancer). The present study reviews issues including causality among tobacco companies’ illegal acts, smoking, and lung cancer occurrence.

**Legal Perspectives**

Epidemiological studies demonstrating that the risk of developing lung cancer is significantly higher for smokers than it is for non-smokers have been consistently presented since the 1950s (Doll and Hill, 1954; Hill et al., 2003). Consequently, smokers who think that they have developed lung cancer due to smoking have raised lawsuits against cigarette firms, demanding compensation for damages (Rutter, 1997). In the case of tobacco lawsuits, the grounds on which plaintiffs claiming damages due to smoking hold defendants legally responsible have tended to develop from the existing responsibility for general unlawful conduct to new arguments in legal principles since the legislation of the Product Liability Act. According to these legal principles, tobacco is a product with defects and plaintiffs have been subjected to damages due to its use; hence, it is possible to hold defendants legally responsible if plaintiffs can only prove the probability of damage occurrence regarding the causal relationship between the defects in tobacco and damage occurrence. This is because unless cigarette firms, which are the defendants, present evidence that they have no involuntary fault, their involuntary fault can be presumed if plaintiffs prove that damages have occurred due to the defects in tobacco. In the Product Liability Act, the defects in a product refer to a state in which that product lacks the safety commonly expected of it in consideration of the characteristics and commonly predicted form of use of the product, the period in which the manufacturer distributed the product, and other conditions related to the product. Consequently, while lawsuits seeking compensation for damages due to products with defects can be classified as those involving product liabilities, it is questionable whether the probability theory of causal relationships or the de facto presumption of involuntary fault in the above arguments constitutes a judicial precedent generally acknowledged in product liability lawsuits.

Plaintiffs argue that because the defects in a product concern safety linked to the risks of damages that the components of the product will incur to life and the body, if tobacco causes diverse diseases including lung cancer in smokers in the long term and makes smoking cessation difficult due to its addictiveness, it is either a product with defects or has such design defects. In addition, manufacturers’ duty to remove risks is imposed broadly from manufacturing to distribution and consists of more than simply informing the consumers about the risks. In other words, because this duty also includes the duty to actively publicize the risks so that the consumers become adequately aware of them, according to this viewpoint, it becomes difficult for cigarette firms to be free from the responsibility of “warning defects” among the product’s defects if they have not fully performed this duty.

When, among a product’s defects, “design defects” are first examined, it is difficult to argue that tobacco sold in the market has failed to reach the levels of safety commonly expected by members of the public with the exception of circumstances such as the inclusion of special substances in tobacco by defendants to increase the product’s addictiveness. Manufacturing defects refer to situations in which a product becomes dangerous because it has been manufactured and processed differently from its originally intended design despite the manufacturer performing the duty of care regarding the manufacturing and processing of that product. Consequently, it is legally difficult to acknowledge the design defects in tobacco based on the product’s health risks and addictiveness. Next, “warning defects” occur when a manufacturer has not faithfully performed the duty of explaining the risks of a product to consumers. In today’s risk society, a manufacturer has a duty to inform and warn consumers about a product so that they can independently and rationally adjust the specific risks of that product and protect themselves. Considering factors including the addictiveness of tobacco and the effect of smoking on adolescents, it is difficult to say that the warning messages on tobacco products distributed in the market such as “Smoking causes diverse diseases including lung cancer and will render even my family and neighbors ill” and “Cigarette smoke contains 2-naphthylamine, nickel, benzene, vinyl chloride, arsenic, and cadmium, which are carcinogenic substances” have an adequate warning effect. This is because there also exist pictorial warning labels, which are more active measures. However, there is much room for legal arguments regarding whether such warning defects have directly caused the development of lung cancer or negative health outcomes in plaintiffs and how much involuntary fault can be ascribed to defendants with respect to such damages.

While judging legal responsibilities for the two types of defects examined above, the question of causal relationships arises. It is possible to calculate the probability of developing lung cancer and the contribution of smoking under specific conditions in the form of statistical figures by entering the characteristics of the injured party such as the duration of smoking, the amount of tobacco smoked, and the age of smoking initiation in epidemiological data. Consequently, plaintiffs argue that when smokers develop lung cancer, it is possible to make de facto presumptions regarding whether smoking has caused cancer based on objective data. In addition, cigarette firms, which are the defendants, have a responsibility to disprove that plaintiffs’ lung cancer has not been caused by smoking. In principle, when specific damages have been incurred due to the defects in a product, the burden of proof regarding that product’s design defects or warning defects lies with plaintiffs, who are the injured party. However, when the causal relationship between specific risk factors and the development of diseases has been scientifically and solidly proven, the burden of proof on the injured party, who are the plaintiffs, may be reduced. This is because when a product is an aggregation of state-of-the-art technology or it is extremely difficult for members of the public other than the experts to detect defects in product liability lawsuits, causal relationships may be presumed for the fair burdening of damages. For example, the trend today is partly to reduce the burden of proof even in judicial precedents regarding pollution lawsuits or medical malpractice lawsuits because demanding strict proof of causal relationships can result in injustice to plaintiffs.

Another point of contention that must be heeded in tobacco lawsuits is defendants’ intent and negligence. Injured patients make an argument for the existence of an involuntary fault in which cigarette firms, as manufacturers of a harmful product, have failed to provide consumers with important information on the product, which should have been publicized, and to perform the duty to warn, which should have been fulfilled to protect consumers’ bodies and lives. This is because an involuntary fault is presumed when damage occurrence in consumers has been proven in a situation where a product has defects. Moreover, injured patients, who are the plaintiffs, argue that intentionality, too, can be acknowledged because cigarette firms have concealed information regarding addictiveness and harmfulness of tobacco while promoting the sales of tobacco by encouraging smoking, improving the taste and aroma of diverse cigarette brands, or increasing the nicotine absorption rate. However, because it is actually difficult to prove intentionality, the question of the de facto presumption of an involuntary fault becomes the point of contention in tobacco lawsuits.

In general, the party whose involuntary fault must be proven is in a state of deliberation where it should have been aware of the occurrence of particular results but has failed to recognize the risks due to its carelessness. In other words, an involuntary fault in tobacco lawsuits consists of a state of deliberation where cigarette firms, which are the defendants, have manufactured tobacco carelessly even though they should have been aware of the possibility of the development of lung cancer in consumers. The problem is that the burden of proof regarding whether defendants have committed an involuntary fault so as to hold them responsible for compensation for damages lies with plaintiffs. Courts’ normative judgment is that defendants have not committed an involuntary fault if and when cigarette firms, which are the defendants, argue that they, too, have been aware of only the general predictability of the development of cancer with respect to the risks of tobacco. However, because it is difficult to directly prove an involuntary fault that is merely under deliberation, when special circumstances or particular circumstantial evidence are judged to exist, it is also possible to presume the involuntary fault of the injured party from such circumstantial evidence in accordance with empirical rules. In addition, the duty to warn regarding the risks of tobacco can be applied more strictly in the case of minors because they possess less ability to judge than adults. However, manufacturers’ responsibility for compensation for damages is reduced through comparative negligence in cases where plaintiffs have voluntarily smoked despite having the knowledge of risks because the reason for imputation also lies with them.

**Epidemiological Perspectives**

In general, the relative risk of lung cancer occurrence is more than 20 times higher for smokers than it is for lifelong non-smokers (Wu-Williams and Samet, 2000). However, tobacco firms argue that because epidemiological evidence for the causality between smoking and lung cancer occurrence is merely a statistical association concerning populations, it cannot serve as evidence for the cause of the outbreak among individual lung cancer patients. In other words, they plead that long-term smoking does not lead to the occurrence of lung cancer in all smokers and that not all lung cancer patients have engaged in smoking. Of course, lung cancer occurrence is also related to factors including outdoor and indoor air pollution, occupational exposure to harmful materials, drinking, and alimentary (dietary) habits. Consequently, defendants, or tobacco firms, argue that plaintiffs must prove that there is a high probability that specific individuals would not have developed lung cancer if they had not smoked. However, to make causal judgments about the causes of disease occurrence, the relationship between the explanatory power of a specific risk factor and inter-individual variations in disease occurrence must be examined. At the same time, this must not be confounded with the magnitude of causal contribution of a specific risk factor to disease occurrence (Davey Smith, 2011). With respect to the causality between smoking and lung cancer, inter-individual variations in disease occurrence can be explained as follows: lung cancer patients also include non-smokers, and only one out of ten smokers develops lung cancer. These points are important grounds for tobacco firms to refute the causal association between smoking and lung cancer at an individual level.

Inter-individual variations in disease occurrence are explained by *heritable factors*, *shared environmental factors*, and *non-shared environmental factors* (Lichtenstein et al., 2000). These factors are different from indices for evaluating the causal contribution of specific factors to disease occurrence, such as the attributable fraction and the population-attributable fraction. If and when heritable factors explain the bulk of inter-individual variations, they are classified as hereditary disorders. However, this does not mean that environmental factors are not involved in the occurrence of hereditary disorders. The percentage to which heritable factors explain inter-individual variations in phenotypes is called heritability, and this is because heritable factors do not signify the power of causal influence over disease occurrence (Burton et al., 2005).

Shared environmental factors can be found in twins who have been raised in the same home as they have been exposed to passive (secondhand) smoking or have similar alimentary habits (Lichtenstein et al., 2000). The power to which an environmental factor not shared by twins explains inter-individual variations in disease occurrence is called the explanatory power of a non-shared environmental factor. According to previous studies that elucidate the degree to which heritable factors, shared environmental factors, and non-shared environmental factors explain inter-individual variations in the occurrence of cancer, non-shared environmental factors exhibit the greatest power in explaining inter-individual variations (Lichtenstein et al., 2000; Plomin, 2011). On the other hand, the power of smoking to explain inter-individual variations in lung cancer has been reported to be approximately 10%, which is relatively high in comparison with other risk factors (Pearce, 2011).

In tobacco lawsuits, lung cancer is not considered as a “specific disease,” where the causes and the results clearly correspond to each other, but as a “non-specific disease,” where diverse causative factors act together to produce the disease. Because the probability of not developing lung cancer is higher than that of developing the disease even when a smoker continues to smoke throughout his or her life (Peto et al., 2000), the risks and causal relationship of smoking in the outbreak of a specific disease in a specific individual are unclear. Consequently, tobacco firms argue that there is no 1:1 association between smoking and lung cancer because of the logic that “B does not exist without A”, which applies to the relationship between the two. However, an association where the causes and the results clearly correspond to each other in a 1:1 ratio, or monocause A that satisfies the necessary and sufficient conditions for the development of disease B cannot exist (Broadbent, 2013). A classification of diseases themselves into specific diseases and non-specific diseases is not easily acceptable to epidemiologists.

While infectious diseases may be considered as “specific diseases” according to traditional definitions, this is not always the case. Although a large proportion of the South Korean population carries the tubercle bacillus (Mycobacterium tuberculosis), the proportion of tuberculosis patients among the total population is very small (Hong et al., 1998). Consequently, the bacteria causing cholera (Vibrio cholerae) and the tubercle bacillus are the necessary conditions for the development of cholera and tuberculosis, respectively. In other words, the attributable fraction is 100% for both the bacteria causing cholera and the tubercle bacillus. One will not develop cholera unless one has been infected with the bacteria causing cholera; and one will not develop tuberculosis unless one has been infected with the tubercle bacillus. However, with the exception of infectious diseases, most non-infectious diseases do not have necessary conditions. This is because risk factors are component causes in non-infectious diseases (Rothman et al., 2008). If “specificity” signifies the magnitude of the causal relationship between risk factors and diseases, as between the bacteria causing cholera and cholera and the tubercle bacillus and tuberculosis, smoking, whose attributable fraction with respect to lung cancer is 90%, can be considered as a very “specific” factor among many other risk factors. In particular, among small-cell lung cancer, squamous cell lung cancer and laryngeal cancer, which are the types of lung cancer that are problematic in tobacco lawsuits today, squamous cell lung cancer exhibits considerable magnitude in such “specificity.”

Epidemiology has played a significant role in proving the causal association between smoking and lung cancer and quantifying the degree of risk of smoking. However, the results of observations, such as individual observations, animal experiments, and experimental analysis of chemical agents, have contributed considerably to the formation of the grounds for the causal association between smoking and lung cancer (Proctor, 2012). The results of the so-called “tobacco juice” animal experiments, which demonstrated the development of tumors by applying the tar in cigarettes to the backs of mice, are representative (Wynder et al., 1953). The results of the chemical analysis of carcinogenic substances in tobacco smoke such as polycyclic aromatic hydrocarbons (PAHs) also help to prove the causality between smoking and lung cancer (Proctor, 2012).

## Conclusions

The present study reviewed issues including causality among tobacco companies’ illegal acts, smoking, and lung cancer occurrence. In tobacco lawsuits so far, the burden of proof regarding negligence and a causal relationship has fallen on plaintiffs, who are the injured party. However, since the legislation of the Product Liability Act, the possibility of mitigating plaintiffs’ burden of proof has opened up. Nevertheless, this alone cannot prevent the immense socioeconomic cost incurred due to smoking. It is legislatively necessary to enact a tobacco management law so that the no-fault liability of tobacco companies, which are the defendants, for compensation can be acknowledged. However, it is necessary to take supplementary measures through the social security system such as establishing the upper limits for liquidated damages in lawsuits and creating a relief fund for the victims of smoking. In addition, it is fundamentally necessary for courts to accept the methods for inferring causality that are based on the natural sciences and epidemiology in situations such as tobacco lawsuits, where a causal relationship cannot be proven easily. In particular, jurists, too, must consider the application of population-based evidence presented by epidemiologists to lawsuits in a forward-looking manner for redressing damages to individuals with diseases; thus, bridging the gap between normative adjudication and scientific judgment to draw a conclusion about a causal relationship. Because epidemiological data are already being used as courtroom evidence to a considerable degree in adjudicating occupational diseases (Bianchi et al., 1999), there is no reason not to use them in tobacco lawsuits as well.

Diverse factors intervene in disease occurrence. In principle, no disease develops due to a single cause. However, the fact that various causes intervene in disease occurrence does not reduce the causal influence of smoking on lung cancer. A very strong cause of lung cancer, smoking, has a greater causal influence than any other factor identified so far and is “specific.” In particular, this is even truer in the case of three carcinomas; small cell lung cancer, squamous cell lung cancer, and squamous cell laryngeal cancer, which are currently being debated in tobacco lawsuits. In epidemiology, Hill’s considerations for causation have been used to determine causality (Porta, 2014). Consequently, when considerations such as biological plausibility, coherence with existing knowledge, experimental demonstration, and analogy have been satisfied through diverse experiments, it is necessary to provide relief to the victims suffering from diseases through the de facto inference of a causal relationship.

The magnitude of causal contribution of a specific risk factor with respect to disease occurrence is expressed in the form of the attributable fraction. The present study examined ways of applying the attributable fraction to population data at an individual level. The attributable fraction can be used to presume the probability of causation because there exists a certain relationship between the attributable fraction and the probability of causation (Broadbent, 2013; Rothman et al., 2008). Consequently, when determining the causality of disease occurrence in individuals in courts, it is necessary to acknowledge the admissibility of evidence held by attributable fraction figures presented by epidemiologists. This is the way to safeguard public sentiments, meet the demands of the times, and render society at large healthy through the regulation of harmful substances.

## Author Contribution Statement

MJ wrote and revised the manuscript.

## Ethical Approval

Not applicable.

## Statement Conflict of Interest

The author disclose no potential conflicts of interest.
